# A New Method of Treating the Fistula Lachrymalis

**Published:** 1781-01

**Authors:** 


II. *A new Method of treating the Fiſtula Lachrymalis. By* Mr. William Blizard, *Surgeon*, F. A. S. (abridged from the Philoſophical Tranſactions, Vol. LXX. Part I.)

THE principal means of cure in the firſt and moſt ſimple ſtage of the *fiſtula lachrymalis*, are, 1ſt, compreſſion; declared by experienced practitioners to be injudicious. 2d, The paſſing an inſtrument into the noſtril and up the duct; a troubleſome and painful operation. The introducing a probe through one of the puncta into the duct; by experience proved to be inadequate to the deſign. 4th, The impelling a fluid by a ſyringe thro' one of the puncta; allowed to be ſometimes uſeful. On reflecting upon this laſt method, Mr. Blizard was induced to think, that if a fluid of a great degree of ſpecific gravity, vity, as quickſilver, could be paſſed through one of the puncta, ſo as to fill the ſac and duct, and preſs upon the obſtructed part, it might reaſonably be expected to remove it, or at leaſt to have a much better chance of doing this than a watery fluid; he therefore provided an inſtrument for the purpoſe; it conſiſts of a fine ſteel pipe, a little curved, cemented in a glaſs tube about ſix inches long: at the top of the tube is a wooden funnel, and at the bottom of this a valve; but he thinks the valve unneceſſary, as the quickſilver may be poured in by an aſſiſtant.

Mr. Blizard has tried this inſtrument in one patient, who had been troubled with a flux of tears and mucus down the cheek from the puncta of the right eye-lids, about ſeven months. The ſteel pipe was paſſed into the inferior punctum without pain or difficulty. The quickſilver was then poured into the funnel, and when it regurgitated out by the ſuperior punctum, the inſtrument was withdrawn. The quickſilver lay in the ſac and duct without exciting pain, about thirty-hours, when it paſſed into the noſe. On the third day the operation was repeated, and upon gently compreſſing the ſac, the greateſt part of the quickſilver and ſome congealed mucus paſſed into the noſtril. After the ſecond or third operation, the ſwelling and diſtenſion of the ſac entirely ſubſided. Upon raiſing the column of quickſilver to twelve inches, it paſſed into the noſe with much greater velocity than before. The patient after this proceſs had been repeated four times, at intervals of a few days, had no diſcharge of mucus, and a tear but very ſeldom, ſo that the parts had a perfectly healthy appearance. Mr. Blizard obſerves, that this operation will perhaps avail only in the firſt or ſimpleſt ſtage of the diſorder, and concludes with remarking that it is ſimple, eaſily executed, productive of but little pain, and attended with no kind of danger.

